# Fossa-Foveolar Mismatch Is Highest in Dysplastic Hips and During External Rotation

**DOI:** 10.1016/j.asmr.2025.101288

**Published:** 2025-10-10

**Authors:** Vera M. Stetzelberger, Cem Cek, Jannine T. Segessenmann, Vlad Popa, Corinne A. Zurmuehle, Joseph M. Schwab, Moritz Tannast

**Affiliations:** a Department of Orthopaedic Surgery and Traumatology, HFR Cantonal Hospital, University of Fribourg, Fribourg, Switzerland; b Department of Orthopaedic Surgery and Traumatology, Inselspital Bern, University of Bern, Bern, Switzerland

## Abstract

**Purpose:**

To identify which hip pathomorphologies and motions are associated with a high fossa-foveolar mismatch (FFM) index.

**Methods:**

Three-dimensional models of hips with femoroacetabular impingement syndrome or developmental dysplasia of the hip (DDH) and control hips were analyzed. Simulations of the physiological range of motion and impingement tests were performed using validated collision detection software. The FFM index—the proportion of the fovea tracking outside the acetabular fossa—was calculated for each motion.

**Results:**

A total of 183 hips with femoroacetabular impingement syndrome and 22 control hips were included. DDH hips showed the highest FFM index (median 0.4), followed by hips with excessive femoral version (median 0.3). Controls had the lowest values (median 0.2). External rotation had the highest FFM in all groups. Motions such as flexion, extension, and abduction had the lowest FFM values.

**Conclusions:**

DDH and excessive femoral version were linked to increased FFM, while hips from the control group presented the lowest mismatch. From all analyzed motions, the highest FFM was found in external rotation in all groups.

**Level of Evidence:**

Level III, retrospective cohort study.

Lesions of the fossa-foveolar-ligamentous complex (LFFC) are remarkably common among young active patients undergoing hip joint–preserving surgery, as more than 9 of 10 patients are affected.^1^^,^^2^ Increasing evidence suggests that lesions of the ligamentum teres (LT) in particular are not merely incidental findings but may be a clinically significant source of persistent intra-articular hip pain, contributing to mechanical symptoms such as instability, clicking, or locking.^3^, ^4^, ^5^, ^6^ Research suggests that those lesions could be associated with adverse outcomes after hip arthroscopy.^7^, ^8^, ^9^, ^10^ The underlying pathomechanisms have not been fully clarified. Klaue et al.^11^ initially suggested that LT degeneration could be due to a pathological positioning of the fovea capitis on the femoral head: the fossa-foveolar mismatch (FFM),^12^ defined by abnormal positioning of the fovea capitis outside the acetabular fossa during specific types of motions. This has been proposed as a possible mechanism for degenerative change around the LT, specifically including impingement of the LT between the lunate surface and the femoral head, as well as abnormal stress concentration at the cartilaginous edge of the acetabular fossa or fovea capitis.^11^^,^^12^ The FFM index was introduced to quantify ligament-cartilage overlap. While it has been shown to be a reliable and reproducible measurement,^12^ little is known regarding its use to identify patient morphologies and specific motions at risk. The purpose of this study was to identify which hip pathomorphologies and motions are associated with a high fossa-foveolar mismatch index. We hypothesized that patients with version abnormalities and developmental dysplasia of the hip (DDH) would present higher FFM indices and that rotation motions would be associated with higher FFM indices.

## Methods

The present 3-dimensional (3D) motion retrospective cohort study was approved by our institutional review board.

### Patients

**Fig 1:**
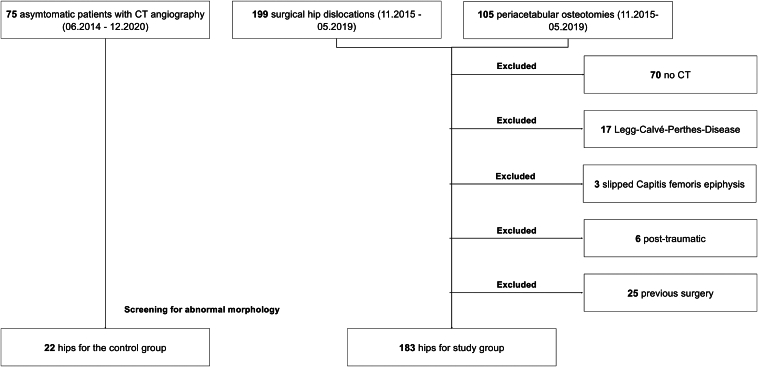
The Consolidated Standards of Reporting Trials flow diagram shows the inclusion and exclusion criteria for the study and control groups.

We assessed consecutive patients who had undergone joint-preserving surgery for femoroacetabular impingement syndrome (FAIS) or DDH at our home institution between November 2015 and May 2019. A total of 304 consecutive patients were evaluated for inclusion, and 183 hips were included in the final analysis (Fig 1; Table 1, Table 2). For the control group, we screened 205 patients who had undergone computed tomography (CT) angiography from June 2014 and December 2020. Twenty-four patients had previous surgery (osteosynthetic material or total hip arthroplasty), and 61 patients had an incomplete CT angiography that we could not use to generate a 3D model. Ninety-eight patients had femoral version pathology (<10 degrees or >25 degrees of femoral anteversion) or acetabular pathology (retroversion or lateral-center edge angle >33° or <22°) and were excluded. Therefore, 22 hips were included in the control group. Standardized preoperative anteroposterior pelvic radiographs were routinely performed in all patients. This allowed us to allocate hips to different study subgroups according to acetabular and femoral morphology using previously established reference values.^13^^,^^18^

**Table 1:** Definition of the 6 Study Subgroups, Describing Distinct Acetabular and Femoral Pathomorphologies, and the Control Group According to Lerch et al^14^

Groups		Definition	Number of Hips
Study subgroups	Dysplasia	LCEA <22° and/or anterior coverage <14%	55
	Overcoverage	LCEA of 34°-39°, not all retroversion signs positive (posterior wall sign, crossover sign, ischial spine sign)	34
	Severe overcoverage	LCEA >39° and/or femoral head touching/crossing the ilioischial line and/or total femoral coverage >93%	23
	Retroversion	Crossover sign,^15^ ischial spine sign,^16^ and posterior wall sign^15^ positive, retroversion index >30%	30
	Deficient version	Femoral version angle <10° according to Murphy et al.^13^	28
	Excessive version	Femoral version angle >25° according to Murphy et al.^13^	123
	Cam-morphology	α angle >50°	29
Control		Asymptomatic hips with CT scan for angiographic diagnostics and normal acetabular and femoral morphology: LCEA 23°-33°, femoral version angle 10°-25° according to Murphy et al.^13^	22

CY, computed tomography; LCE, lateral center-edge angle.

**Table 2:** Demographic and Radiologic Parameters of the Study Group (Consisting of 4 Acetabular and 3 Femoral Subgroups) and the Control Group (Consisting of 22 Asymptomatic Patients With a CT Scan for Angiography Diagnostics)

		Study Subgroups (n = 183)							Control Group (n = 22)	*P* Value
		Acetabular Morphology				Femoral Morphology				
Variables	Overall Study Group	Dysplasia (n = 55)	Overcoverage (n = 34)	Severe overcoverage (n = 23)	Retroversion (n = 30)	Cam-morphology (n = 29)	Deficient Femoral Version (n = 28)	Excessive Femoral Version (n = 123)		
Demographics										
Number of hips (patients)	183 (155)	55 (45)	34 (33)	23 (21)	30 (30)	29 (29)	28 (24)	123 (104)	22 (22)	
Age at imaging, y	28 ± 8 (15-54)	30 ± 8 (18-50)	26 ± 9 (16-54)	29 ± 8 (18-50)	25 ± 6 (16-36)	28 ± 8 (18-50)	28 ± 9 (15-54)	27 ± 8 (16-50)	55 ± 9 (37-66)	<.001
Sex (% men)	38 (20)	14 (26)	9 (26)	3 (13)	9 (30)	6 (21)	11 (39)	14 (11)	16 (73)	<.001
Side (% right)	110 (59)	36 (66)	20 (59)	11 (48)	18 (60)	23 (79)	16 (57)	75 (61)	12 (55)	.699
BMI	24 ± 5 (17-39)	25 ± 5 (18-39)	25 ± 4 (19-36)	25 ± 6 (17-34)	24 ± 5 (17-34)	24 ± 4 (19-33)	27 ± 4 (20-36)	24 ± 4 (17-39)	27 ± 4 (19-33)	.04
Radiological										
LCEA, °	28 ± 12 (–17 to 64)	15 ± 6 (–17 to 22)	36 ± 2 (34-39)	47 ± 6 (40-61)	31 ± 9 (9-51)	28 ± 6 (10-38)	33 ± 7 (19-55)	27 ± 11 (17-61)	33 ± 4 (25-39)	<.001
Extrusion index, %	22 ± 11 (–6 to 67)	34 ± 6 (22-67)	15 ± 2 (10-18)	6 ± 6 (–4 to 17)	19 ± 8 (3-41)	22 ± 6 (11-39)	17 ± 6 (–4 to 29)	23 ± 11 (–4 to 67)	18 ± 4 (11-26)	<.001
Acetabular index, %	5 ± 9 (–20 to 31)	14 ± 6 (2-31)	1 ± 5 (–12 to 11)	-7 ± 5 (–14 to 5)	2 ± 7 (–11 to 19)	5 ± 8 (–19 to 20)	4 ± 5 (–11 to 13)	5 ± 10 (19-31)	3 ± 5 (–6 to 9)	<.001
Crossover sign, % positive	148 (79)	38 (69)	32 (94)	19 (83)	30 (100)	21 (72)	23 (82)	97 (79)	17 (77)	.04
PW sign, % positive	121 (65)	45 (82)	21 (62)	10 (44)	30 (100)	22 (76)	17 (61)	75 (61)	14 (64)	.001
Ischial spine sign, % positive	84 (45)	21 (38)	19 (56)	12 (52)	26 (87)	15 (52)	10 (37)	50 (41)	0 (0)	<.001
Retroversion index, %	15 ± 16 (0-77)	10 ± 13 (0-54)	23 ± 19 (0-77)	21 ± 16 (0-53)	42 ± 10 (31-77)	17 ± 19 (0-49)	12 ± 17 (0-77)	15 ± 1 (0-57)	18 ± 22 (0-70)	<.001
TAC, %	77 ± 12 (32-100)	64 ± 7 (32-76)	84 ± 5 (74-93)	94 ± 7 (77-100)	78 ± 10 (58-100)	77 ± 7 (65-93)	82 ± 9 (67-100)	76 ± 11 (32-100)	83 ± 7 (62-94)	<.001
TPC, %	44 ± 11 (10-83)	37 ± 9 (16-55)	46 ± 11 (10-66)	54 ± 12 (35-77)	36 ± 9 (16-61)	43 ± 10 (21-61)	48 ± 10 (29-74)	44 ± 11 (10-77)	50 ± 8 (32-63)	<.001
CCD, angle, °	137 ± 8 (117-161)	138 ± 8 (126-157)	140 ± 8 (129-161)	134 ± 3 (128-139)	136 ± 8 (120-158)	135 ± 6 (126-158)	134 ± 8 (117-146)	138 ± 7 (121-161)	134 ± 5 (124-140)	.07
α angle, °	53 ± 13 (30-120)	54 ± 13 (34-120)	50 ± 10 (36-75)	44 ± 9 (31-73)	52 ± 11 (30-79)	62 ± 10 (52-100)	54 ± 12 (31-75)	53 ± 14 (30-120)		.002
Femoral version, °	33 ± 18 (–11 to 72)	36 ± 16 (–5 to 68)	44 ± 9 (25-63)	44 ± 7 (34-58)	32 ± 18 (0-68)	33 ± 15 (–4 to 57)	1 ± 5 (–11 to 8)	43 ± 9 (26-68)	20 ± 5 (10-28)	<.001

NOTE. The sum of all hips in the 7 study groups exceeds 183, as one hip can be allocated to several groups. Femoral version was measured using the Murphy method.^17^ Continuous values are given as mean ± standard deviation with range in parentheses. Categorical values are given as absolute numbers with percentages in parentheses. Continuous variables were compared with the analysis of variance (parametric variables) or Kruskal-Wallis (nonparametric) test, and the significance level was adjusted according to the Bonferroni correction for 8 groups with an α level of .05 (.05/8 = .006). Categorical variables were compared using the χ^2^ test.

BMI, body mass index; CCD, caput-collum-diaphyseal; LCEA, lateral center-edge angle; PW, posterior wall; TAC, total anterior coverage; TPC, total posterior coverage.

We established a control group by selecting 22 asymptomatic patients who had undergone a CT scan of the pelvis and lower extremity for angiography at our institution between June 2014 and December 2020. A normal acetabular and femoral morphology was required for all patients of the control group.^13^^,^^18^

Continuous values are given as median (range). The groups were compared using the Kruskal-Wallis test. Specific comparison between each study subgroup and the control group is significant (Wilcoxon test). Patients could be allocated to different pathological groups simultaneously.

### Three-Dimensional Model

**Fig 2:**
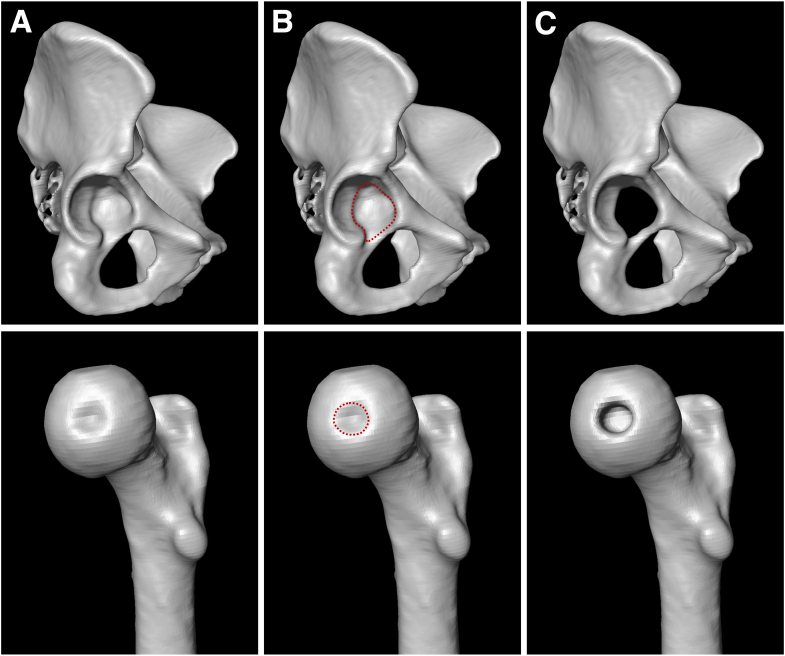
(A) We built a 3-dimensional model from preoperative computed tomography scans of each patient. (B) The fossa and fovea are identified and marked. (C) The fossa is excised out of the acetabulum, and the fovea is manually recessed by approximately 10 mm.

We built a 3D surface model for all hips using the semi-automatic segmentation software AMIRA (Thermo Fisher Scientific). The borders of the acetabular fossa were carefully identified so that the entire acetabular fossa could be excised. Likewise, the borders of the fovea capitis were carefully identified so that the fovea could be manually recessed by approximately 10 mm (Fig 2A-C).

### Fossa-Foveolar Mismatch for Different Morphologies and Motions

**Fig 3:**
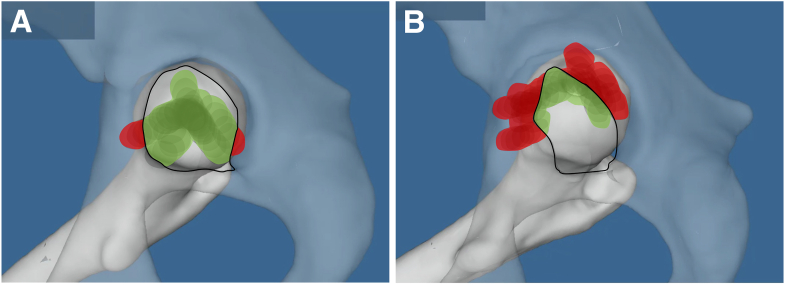
The fossa-foveolar mismatch is calculated by dividing the surface area of the tracking pattern located outside the fossa by the total surface area. (A) This patient from the control group presents a low fossa-foveolar mismatch (FFM) index. (B) This patient from the dysplastic group presents a high FFM index.

Physiological range of motion was simulated with a validated 3D collision detection software.^19^^,^^20^ We analyzed 6 aspects of 3 single-plane motions (flexion, extension, abduction, adduction, internal rotation, and external rotation) and 5 composite motions: internal and external rotation in 90° of flexion, internal rotation in 90° flexion and 20° adduction (simulating the anterior impingement test), and external rotation in 20° extension and 20° abduction (simulating the posterior impingement test). The surface area generated by the tracking pattern of the fovea capitis was measured for each simple and composite motion. For that purpose, transparency of the pelvis was set at 50% to better visualize the position of the fovea when it was outside of the acetabular fossa (Fig 3 A and B). The FFM index was calculated for each motion, utilizing the previously described technique, by dividing the surface area of the tracking pattern located outside of the fossa by the total surface area.^17^^,^^21^ In addition, all the measured foveolar tracking patterns for that hip were overlaid on each other, and an “overall” FFM index was calculated.

### Statistical Analysis

Normal distribution for continuous variables was tested using the Kolmogorov-Smirnov test. Demographic continuous variables were compared using the analysis of variance test. The “overall” FFM indices for all study groups were compared using the Kruskal-Wallis test. Each pathological morphology study group was compared to the control group using the Wilcoxon test. The FFM indices for each specific motion were compared using the Kruskal-Wallis test. Each study morphology study group was compared to the control group using the Wilcoxon test. Significance levels for the variables compared using analysis of variance and Kruskal-Wallis tests were adjusted according to the Bonferroni correction for 8 groups, with an α level of .05 (.05/8 = .006).

## Results

### Fossa-Foveolar Mismatch According to Different Hip Morphologies

**Fig 4:**
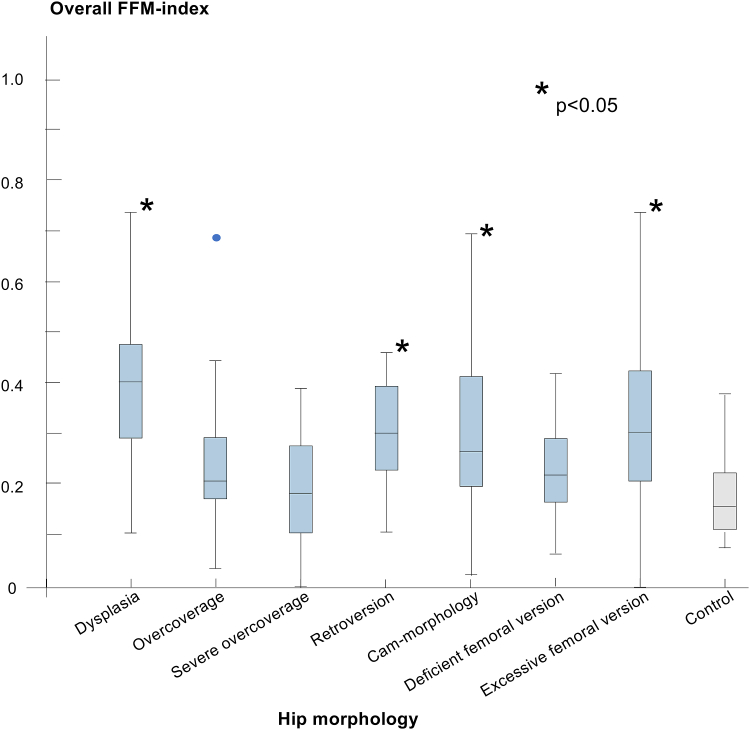
The boxplot displays the overall fossa-foveolar mismatch index for each study subgroup (blue) compared to the control group (gray). ∗Significantly different from the control group.

The median “overall” FFM index for the control group was 0.2 (range, 0.1-0.4). Compared to the control group, the dysplasia, acetabular retroversion, excessive femoral version, and cam groups had higher median “overall” FFM indices of 0.4 (range, 01.-0.7), 0.3 (0.1-0.7), 0.3 (0.1-0.7), and 0.27 (0.03-0.7), respectively (*P* < .001; Appendix Table 1, available at www.arthroscopyjournal.org). Patients with overcoverage, severe overcoverage, or deficient femoral version had FFM indices similar to the control group. In contrast, patients with dysplasia, acetabular retroversion, or excessive femoral version showed significantly higher FFM indices than in the control group (Fig 4).

### Fossa-Foveolar Mismatch of Each Specific Motion

**Fig 5:**
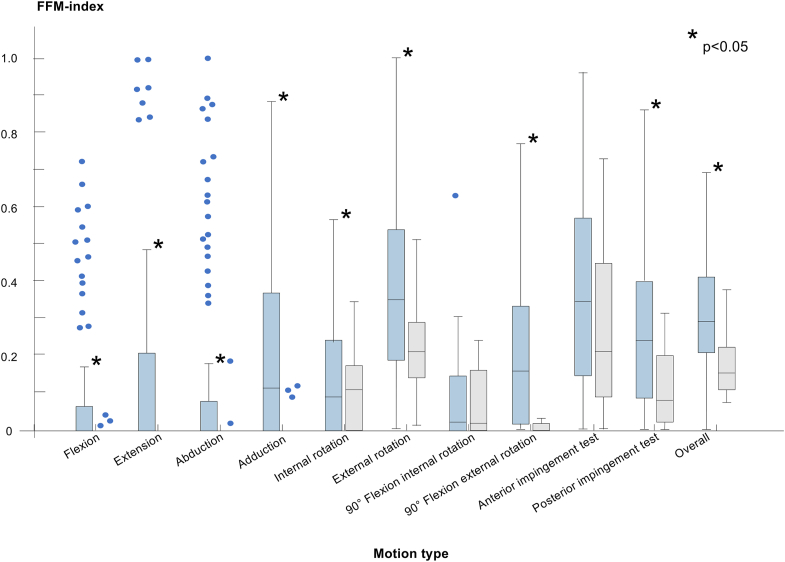
The boxplot displays the fossa-foveolar mismatch index for each motion in the study group (blue) and the control group (gray). ∗Significantly different from the control group.

The FFM index was highest in external rotation in both the control and study groups, with a median of 0.2 (0.01-0.5) and 0.3 (0-1), respectively (Appendix Table 1, available at www.arthroscopyjournal.org). Among all assessed motions, only internal rotation at 90° of flexion and the anterior impingement test showed comparable results between the study and control groups (Fig 5). In our sex-based subanalysis of the study group, FFM indices were comparable between males and females in most motions. However, males had higher values in internal rotation and internal rotation at 90° of hip flexion (*P* = .003 and .04, respectively; Table 3).

**Table 3:** The Fossa-Foveolar Mismatch According to Sex for All Analyzed Motions

Motion	Female	Male	*P* Value
Flexion index	0.0 (0.0-0.72)	0.01 (0.0-0.66)	.5
Extension index	0.0 (0.0-1.0)	0.0 (0.0-0.99)	.77
Abduction index	0.0 (0.0-1.0)	0.0 (0.0-1.0)	.66
Adduction index	0.11 (0.0-1.0)	0.13 (0.0-0.67)	.71
Internal rotation index	0.05 (0.0-0.92)	0.16 (0.0-0.64)	.003
External rotation index	0.35 (0.0-1.0)	0.32 (0.01-0.63)	0.32
90° of flexion internal rotation index	0.01 (0.0-0.48)	0.08 (0.0-0.85)	0.04
90° of flexion external rotation index	0.17 (0.0-0.77)	0.08 (0.0-1.0)	0.32
Anterior impingement test index	0.32 (0.0-0.89)	0.44 (0.0-0.69)	0.05
Posterior impingement test index	0.25 (0.0-1.0)	0.21 (0.0-0.89)	0.64
Total range of motion index	0.29 (0.0-0.91)	0.30 (0.1-0.69)	0.14

## Discussion

In our study, we observed the highest FFM indices in DDH and excessive femoral version. Hips in the control group in our study had the lowest FFM index of all the groups. In terms of specific motions, we observed the highest FFM index values for external rotation and internal rotation in 90° of flexion with 20° of adduction (anterior impingement test) across all groups.

This study aimed to identify specific hip morphologies and motions at particular risk for a high FFM index. This may provide additional information about the relationship between these morphologies and a hypothesized pathomechanism for degenerative changes to the LT and surrounding structures: the fossa-foveolar mismatch. While most studies have focused on peripheral cartilage and labral lesions, degeneration of the central aspects of the joint (the LFFC) is gaining increased attention due to its high incidence.^1^^,^^22^, ^23^, ^24^, ^25^, ^26^, ^27^ Specifically, clinical and histologic studies suggest that lesions of the LT may play a role in persistent intra-articular hip pain.^3^, ^4^, ^5^^,^^28^ Also, LT damage was identified as an independent factor associated with poorer postoperative outcomes after hip arthroscopy.^7^^,^^8^ The cause for those lesions, however, has not been clearly determined.

### Fossa-Foveolar Mismatch According to Different Hip Morphologies

The control group displayed the smallest overall FFM index, with a median of 0.16 (0.08-0.44). In a normal hip, the fovea capitis remains mostly confined to the acetabular fossa throughout the entire range of motion we tested. By remaining within the fossa, it avoids overlap with the lunate cartilage.

While hips with cam morphology had higher FFM indices than the control hips, values were lower than in the overall study group (with a median of 0.27 compared to 0.27). Specifically, patients with dysplasia displayed a higher FFM (median of 0.40). These observations go in line with the findings of Zhang et al.,^29^ who found that 47% of patients with borderline DDH had an LT tear prevalence of 47%, whereas patients with cam-type FAIS without DDH had a lower prevalence of 25%. Patients with dysplasia exhibited the highest FFM index, which may be attributable to several underlying factors. First, it could be due to a wider fovea in dysplastic compared to normal hips. Nötzli et al.^30^ quantified the width of the fovea on anteroposterior pelvic radiographs by calculating the “fovea angle,” with dysplastic hips displaying significantly larger fovea angles compared to nondysplastic hips. Second, the fovea is located more superiorly on the femoral head in dysplasia (“fovea alta”), sometimes even extending beyond the borders of the fossa.

Domb et al.,^31^ reporting a series of 16 patients (17 hips) with hip dysplasia undergoing periacetabular osteotomy and concomitant hip arthroscopy, identified lesions of the LT in 88% (15/17) of hips. Perumal et al.^10^ reported on over 1,935 hip arthroscopies across a variety of hip pathologies and indicated an overall incidence of LT lesions in 17% of hips (323/1935). While they did not specifically report the incidence of LT lesions in hips with dysplasia, they did note a statistically significant difference in lateral-center edge angle between patients with LT lesions (lower) than without (higher), which would be consistent with our study results. These findings, considering the results of our study, support the need for more robust clinical reporting on the incidence of LT, foveal, and acetabular fossa lesions in specific patient populations. This reporting may help provide additional clinical context for the hypothesized FFM pathomechanism.

Several studies have identified specific acetabular morphologies and FAIS types associated with LT lesions. Acetabular undercoverage, commonly seen in borderline DDH, has been consistently linked to higher rates of LT tears.^5^^,^^29^^,^^32^ Zhang et al.^29^ found a 47% prevalence of LT tears in patients with borderline DDH compared to 25% in patients with cam-type FAIS, suggesting that undercoverage places increased stress on the LT. Similarly, Lodhia et al.^33^ identified central acetabular osteophytes—often indicative of altered joint loading—as a factor associated with LT pathology. Kaya et al.^26^ and Lee et al.^34^ also showed that cam-type FAIS is associated with LT lesions, particularly when accompanied by chondral damage on the femoral head or acetabulum. These findings suggest that both undercoverage and impingement morphologies can compromise LT integrity, although the mechanisms may differ—instability in the former and mechanical overload in the latter.

### Fossa-Foveolar Mismatch of Each Specific Motion

The foveolar tracking patterns during external rotation and anterior impingement had the highest FFM index in all groups. Specifically, in dysplastic hips, the FFM index for external rotation was measured at 0.52, indicating that over 50% of the tracking pattern occurred outside the confines of the acetabular fossa. Acute twisting motion of the hip into external rotation has been reported as a cause of ligamentum teres injury.^3^^,^^35^^,^^36^ While this has been attributed to the fact that the LT is elongated during external rotation, making it particularly vulnerable to mechanical strain,^3^^,^^37^ this may not be the only explanation. This motion is particularly common in athletes such as ballet dancers^38^ and gymnasts,^39^ who regularly engage in extreme ranges of motion but do not necessarily perform these motions in a forceful way.

### Limitations

The present study has limitations. First, we used a collision detection software that calculates impingement zones based on the osseous morphology exclusively. This method does not account for soft tissue constraints, which can significantly influence range of motion and, consequently, the FFM. The absence of soft tissue considerations in our analysis may have led to an overestimation of the range of motion and calculated FFM. Nevertheless, our software has been employed in multiple previous studies to simulate range of motion and impingement scenarios, providing consistent and reproducible results. Importantly, this method has also been applied to hips with various conditions, including dysplastic hips,^14^, ^40^, ^41^ hips with high femoral version,^42^ and post–Legg-Calvé-Perthes disease deformities,^43^ supporting its robustness across different morphologies. Furthermore, as all patients were analyzed using the same modeling protocol, the comparisons between groups remain valid.

Second, we did not test all combination motions, such as flexion with external rotation and extension with internal rotation (necessary for squatting), which are considered to put tension on the ligament.^37^^,^^44^ Third, the explanation for lesions of the LFFC remains theoretical at this stage. Fourth, our analysis utilized a 2-dimensional assessment of a complex 3D process, inherently simplifying the spatial complexity of the phenomenon. Nevertheless, our method has been previously validated and shown to be highly reliable and reproducible.^12^ This simplification should therefore not impact the overall robustness of our findings.

## Conclusions

DDH and excessive femoral version were linked to increased FFM while hips from the control group presented the lowest mismatch. From all analyzed motions, the highest FFM was found in external rotation in all groups.

## Disclosures

The authors declare the following financial interests/personal relationships which may be considered as potential competing interests: V.M.S. has received funding grants and financial support from the Swiss Society of Orthopaedics and Traumatology Swiss Orthopaedics. J.M.S. is a consultant or advisor for DePuy Synthes, Mathys, and Lima. M.T. has received speaking and lecture fees from AO Trauma and is a consultant or advisor for DePuy Synthes, Mathys, and Lima. All other authors (C.C., J.T.S., V.P., C.A.Z.) declare that they have no known competing financial interests or personal relationships that could have appeared to influence the work reported in this paper.
