# Supplementary material for: Fossa-Foveolar Mismatch Is Highest in Dysplastic Hips and During External Rotation
Source: Arthrosc Sports Med Rehabil. 2025 Oct 10;7(6):101288. doi: 10.1016/j.asmr.2025.101288 (PMC12800802; doi:10.1016/j.asmr.2025.101288)
Supplement: Supplemental table 1 [file mmc1.docx]

**Supplemental table 1:** The fossa-foveolar mismatch in each study sub- and control group for all analyzed motions

|  |  |  | Study subgroups (n = 183) | | | | | |  | Control group (n = 22) |  |
| --- | --- | --- | --- | --- | --- | --- | --- | --- | --- | --- | --- |
|  |  |  | Acetabular morphology | | | | Femoral morphology | | |  | p-Value |
| Motion type | Variables | Overall study group | Dysplasia (n = 55) | Overcoverage (n = 34) | Severe overcoverage (n = 23) | Retroversion (n = 30) | Deficient version (n = 28) | Excessive version (n = 123) | Cam-morphology (n = 29) |  |  |
| **simple** | **Flexion index** | 0 (0-0.72) | 0.03 (0-0.66) [^a^](#tbtablefn1) | 0.01 (0-0.51) | 0.02 (0-0.69) | 0.01 (0-0.54) [^a^](#tbtablefn1) | 0 (0-0.72) | 0.02 (0-0.66) [^a^](#tbtablefn1) | 0 (0-0.54) [^a^](#tbtablefn1) | 0 (0-0.04) | 0.0028 |
|  | **Extension index** | 0 (0-1) | 0.21 (0-1) [^a^](#tbtablefn1) | 0 (0-0.75) [^a^](#tbtablefn1) | 0.18 (0-1) | 0 (0-0.92) [^a^](#tbtablefn1) | 0 (0-0.71) | 0.06 (0-1) [^a^](#tbtablefn1) | 0 (0-1) [^a^](#tbtablefn1) | 0 (0-0) | 0.0001 |
|  | **Abduction index** | 0 (0-1) | 0.04 (0-1) [^a^](#tbtablefn1) | 0 (0-0.83) | 0.03 (0-0.80) | 0 (0-0.67) | 0 (0-0.52) | 0 (0-1) [^a^](#tbtablefn1) | 0.00 (0.00-0.74) | 0 (0-0.19) | 0.0294 |
|  | **Adduction index** | 0.11 (0-1) | 0.41 (0-1) [^a^](#tbtablefn1) | 0.07 (0-0.85) [^a^](#tbtablefn1) | 0.24 (0-1) | 0.1 (0-1) [^a^](#tbtablefn1) | 0.01 (0-0.80) [^a^](#tbtablefn1) | 0.19 (0-1) [^a^](#tbtablefn1) | 0.13 (0.00-1) [^a^](#tbtablefn1) | 0 (0-0.12) | <0.0001 |
|  | **Internal rotation index** | 0.09 (0-0.92) | 0.19 (0-0.71) | 0.01 (0-0.71) | 0 (0-0.69) | 0.14 (0-0.52) | 0.21 (0-0.92) [^a^](#tbtablefn1) | 0.02 (0-0.71) | 0.02 (0.00 -0.69) | 0.11 (0-0.48) | 0.8469 |
|  | **External rotation index** | 0.35 (0-1) | 0.52 (0-1) [^a^](#tbtablefn1) | 0.22 (0-0.93) | 0.54 (0-1) | 0.28 (0-1) [^a^](#tbtablefn1) | 0.17 (0-0.47) | 0.44 (0-1) [^a^](#tbtablefn1) | 0 (0 - 1) | 0.21 (0.01-0.51) | 0.0082 |
|  | **90°flexion internal rotation index** | 0.02 (0-0.63) | 0.09 (0-0.46) | 0 (0-0.27) | 0 (0-0.33)xs | 0.04 (0-0.51) | 0.05 (0-0.63) | 0 (0-0.51) | 0 (0 - 0.57) | 0.02 (0-0.45) | 0.7531 |
|  | **90° flexion external rotation index** | 0.16 (0-0.92) | 0.24 (0-0.92) [^a^](#tbtablefn1) | 0.18 (0-0.61) | 0.25 (0-0.82) [^a^](#tbtablefn1) | 0.12 (0-0.92) [^a^](#tbtablefn1) | 0 (0-0.36) [^a^](#tbtablefn1) | 0.23 (0-0.92) [^a^](#tbtablefn1) | 0.17 (0 - 0.92) [^a^](#tbtablefn1) | 0 (0-0.27) | <0.0001 |
| **Composite** | **Anterior impingement test index** | 0.34 (0-0.96) | 0.56 (0-0.96) | 0.21 (0-0.76) | 0.18 (0-0.91) | 0.38 (0-0.96) | 0.29 (0-0.87) | 0.33 (0-0.96) | 0.27 (0 - 0.96) | 0.21 (0-0.73) | 0.2919 |
|  | **Posterior impingement test index** | 0.24 (0-1) | 0.36 (0-1) | 0.21 (0-0.75) | 0.45 (0-0.90) | 0.23 (0-0.85) | 0.06 (0-0.34) | 0.32 (0-1) | 0.24 (0 - 0.86) [^a^](#tbtablefn1) | 0.08 (0-0.32) | 0.0008 |
|  | **Total range of motion index** | 0.29 (0-0.91) | 0.4 (0.11-0.74) [^a^](#tbtablefn1) | 0.26 (0-0.91) | 0.28 (0.03-0.76) | 0.3 (0.11-0.67) [^a^](#tbtablefn1) | 0.22 (0.07-0.50) | 0.31 (0-0.91) [^a^](#tbtablefn1) | 0.27 (0.03-0.69) [^a^](#tbtablefn1) | 0.16 (0.08-0.44) | <0.0001 |

<TAB-FN>^a^significantly different from the control group.

<TAB-FN>Continuous variables were compared with the ANOVA (parametric variables) or Kruskal-Wallis (non-parametric) test and the significance level was adjusted according to the Bonferroni correction for 8 groups with an alpha level of 0.05 (0.05/8 = 0.006)
